# Micro-computed tomography enables rapid surgical margin assessment during breast conserving surgery (BCS): correlation of whole BCS micro-CT readings to final histopathology

**DOI:** 10.1007/s10549-018-4951-3

**Published:** 2018-09-17

**Authors:** David M. McClatchy, Rebecca A. Zuurbier, Wendy A. Wells, Keith D. Paulsen, Brian W. Pogue

**Affiliations:** 1 Thayer School of Engineering, Dartmouth College, 14 Engineering Drive, Hanover, NH 03755 USA; 2 Department of Radiology, Geisel School of Medicine, Dartmouth College, 1 Rope Ferry Road, Hanover, NH 03755 USA; 3 Department of Pathology, Geisel School of Medicine, Dartmouth College, 1 Rope Ferry Road, Hanover, NH 03755 USA; 4 Norris Cotton Cancer Center, Geisel School of Medicine, Dartmouth College, 1 Rope Ferry Road, Hanover, NH 03755 USA; 5 Present Address: Department of Radiation Oncology, Massachusetts General Hospital, 5 Fruit Street, Boston, MA 02114 USA

**Keywords:** Breast conserving surgery, Micro-computed tomography, Surgical margin assessment, Intraoperative

## Abstract

**Background:**

Roughly 23% of breast conserving surgery (BCS) patients undergo a second re-excision procedure due to pathologically positive surgical margins. We investigated the feasibility and potential value of micro-Computed Tomography (micro-CT) as a surgical margin guidance tool during BCS.

**Methods:**

A cohort of 32 BCS specimens was prospectively imaged with a pre-clinical micro-CT system upon arrival in the surgical pathology laboratory. Reconstructed micro-CT scans were evaluated retrospectively by an experienced breast radiologist, who provided binary determinations whether lesions extended to the specimen margin. These readings were then compared to the final pathological diagnosis and to 2D specimen radiography readings.

**Results:**

Of the 32 specimens imaged, 28 had malignant and four had benign pathological diagnoses. Overall five (four malignant, one benign) of the 32 specimens had lesion tissue extending to the margin. For all 32 specimens, micro-CT reconstructions were calculated (< 4 min. acquisition + reconstruction time) and each specimen was volumetrically analyzed by a radiologist. Of the 28 malignant specimen readings, 18 matched the final pathological diagnosis [64%, 95 CI (47%–81%)], with a negative predictive value of 89% [95 CI (74%–96%)]. Micro-CT readings revealed changes in the tumor location and margin status as compared to single-projection radiography readings.

**Conclusions:**

Micro-CT scanning of BCS specimens enabled margin status assessment over the entirety of the surgical surface in a clinically relevant time frame, provided additional spatial information over single-projection radiography, and may be a potentially useful BCS guidance tool.

**Electronic supplementary material:**

The online version of this article (10.1007/s10549-018-4951-3) contains supplementary material, which is available to authorized users.

## Background

Breast conserving surgery (BCS) in combination with radiotherapy has become standard-of-care for treatment of early stage breast cancer, offering equivalent survival to full mastectomy, but with a far less extensive surgical procedure [1]. However, this equivalency holds true only if resected specimens have negative margins [2, 3]. In non-palpable cancers, BCS guidance involves pre-operative wire placement [4, 5] or radioactive seed tumor localization [6], often followed by specimen mammography [7, 8]. Yet, 20–40% of patients who undergo BCS require re-excision because of a pathologically positive or close margin [9]. Additionally, substantial variability exists among surgeons and institutions, with a reported re-excision rates in the range of 0–70% and 1.7–20.9%, respectively [9, 10], highlighting the clinical need for better surgical guidance during BCS procedures.

The pre-clinical imaging technology, micro-Computed Tomography (micro-CT), offers three dimensional (3D) image reconstruction with sub-millimeter resolution but at much smaller fields of view than clinical CT. While this technology has been utilized extensively in pre-clinical specimen and animal studies [11–13], it has not been widely used to image surgically resected breast tissues [14]. Tang et al. have motivated the potential value of this technology in assessing shaved cavity margins [15] and they have also shown it to measure the largest tumor dimension in resected BCS specimens accurately [16]. However, the diagnostic value of micro-CT in assessing whole resected BCS specimens has not been investigated. Accordingly, the feasibility and potential diagnostic value of micro-CT was evaluated as a surgical guidance tool for BCS by performing an initial correlation of micro-CT analysis to the final histopathologic diagnosis, and qualitatively comparing specimen micro-CT to single-projection specimen mammography.

## Materials and methods

### Overview of specimen imaging protocol

From May of 2017 to January of 2018, a cohort of 32 BCS specimens was imaged prospectively under a HIPPA compliant, Institutional Review Board (IRB) approved observational protocol. Patients undergoing consented and elective BCS were considered for this study. After each specimen was surgically removed and inked to record its orientation [17], it was placed on a radiolucent grid (AccuGrid, Beekley Medical, Bristol, CT), sealed in a plastic bag, and underwent single-projection specimen mammography to confirm the primary lesion and surgical clip were removed from the patient. The sealed specimen was then sent to the Pathology specimen grossing laboratory. Upon arrival, each specimen was removed from the plastic bag, then scanned with the micro-CT in the same orientation as single-projection specimen mammography, and subsequently returned to the pathologists’ assistant for standard gross analysis and histological processing. Only single-projection specimen mammography was available for this study. Micro-CT scans were analyzed retrospectively by an experienced breast radiologist (R.A.Z) blinded to the pathological diagnosis. Participant consent was waived by the IRB because the study did not interfere with standard-of-care nor did it disclose or analyze protected health information (PHI).

### Micro-CT imaging device and reconstructions

Upon arrival in Pathology, specimens were compressed between two acrylic plates and imaged with a micro-CT scanner (IVIS SpectrumCT, PN 128,201, PerkinElmer, Hopkington, MA), which was physically located in the specimen grossing laboratory in order to minimize tissue handling time. Scanning was performed at X-ray settings of 50 kVp and 1 mA with a 440 µm aluminum filter. The X-ray source-detector pair was fixed, with a 50 µm focal spot size. The specimen was rotated through 360° and received a 100 ms exposure at each 0.5° increment (72 s acquisition). Standard filtered back projection (FBP) was used to reconstruct an image volume of 12 × 12 × 3 cm^3^ with 0.15 mm cubic voxels (~ 2 min. reconstruction time). The X-ray detector (3072 × 864 pixels) was binned down by a factor of 4 (1024 × 216 pixels) in order to increase the signal to noise ratio (SNR) and decrease the total scan time. A previous publication reported the micro-CT to have a resolution of 240 µm and an SNR of 34 for this acquisition setting [18]. While the system is capable of capturing a voxel size of 40 µm, the exposure and reconstruction times would have to be increased to non-clinically relevant timescales if scanning the entire field of view. For selected scans, 3D visualization models were created using the “Volume Rendering” tool in 3D Slicer (v4, https://www.slicer.org) [19].

### Pathological diagnosis

After micro-CT scanning, specimens were returned to the pathologists’ assistants, who performed standard-of-care gross analysis to determine both representative sections of lesional tissue as well as all areas of possible lesion involvement with the specimen margin. These tissue sections underwent standard histological processing to create hematoxylin and eosin (H&E) stained slides, which were diagnosed by an experienced histopathologist (W.A.W.). From these slides, distances from lesion involvement to each margin were measured. Tabulated diagnoses and distances associated with each margin of each specimen are included in Supplemental File 1.

### Micro-CT image interpretation

After scans were acquired, an experienced breast radiologist (R.A.Z.) retrospectively analyzed the scans, which were sequentially (scan-mode) presented in two orthogonal views. Features such as speculated masses, distorted tissue architecture, and clustered micro-calcifications were evaluated to yield a binary reading of positive or negative margins. The radiologist was blinded to the final pathological diagnosis as well as the pathological diagnosis of the pre-operative needle core biopsy. For four specimens, micro-CT scans were compared retrospectively to de-identified single-projection specimen mammograms to view qualitatively the effect of micro-CT reconstruction on overlapping structures in the projection images.

### Statistical methods

All statistical analyses were performed using MATLAB (v2016a, Mathworks, Natick, MA). For reader performance metrics, confidence intervals were calculated using a binomial distribution. A one-sided Fisher’s exact test was used to calculate statistical significance between observed involvement in the different surface planes.

## Results

### Specimen characteristics

Specimen characteristics and associated margin status are compiled in Table 1. For this study, a pathologically positive margin, defined as malignant or benign lesional tissue extending to the specimen surface, was the measured end-point determined by histopathology. Of 32 specimens, 16% (5/32) had a pathologically positive margin, and of those, 80% (4/5) were malignant (or 13%, 4/32 of all specimens). However, the actual re-excision rate is likely be higher as a close (≤ 2 mm) DCIS margin may result in re-excision depending on patient characteristics and physician preference [3, 20]. In-situ cancer was more likely to be present with invasive cancer (13/32) than to appear on its own (3/32) (*p* < 0.005), and conversely, invasive cancer was just as likely to be present with in-situ disease (13/32) as to occur on its own (12/32) (*p* = 0.5).

**Table 1:** Pathological diagnoses of 32 BCS specimens

	N *(w/wire, w/o wire)*	Margin status		
		Pathologically positive 0 mm	Pathologically negative “Close” > 0 mm and ≤ 2 mm	Pathologically negative “Distant” > 2 mm
**Invasive only**	**12** (*9*,*3*)	**1**	**3**	**8**
Ductal carcinoma	4	0	0	4
Lobular carcinoma	4	1	0	3
Mucinous	2	0	2	0
Ductal and lobular	2	0	1	1
**In-situ only**	**3** (*2*,*1*)	1	1	1
DCIS	2	0	1	1
Pleomorphic LCIS	1	1	0	0
**Invasive + In-Situ**	**13** (*8,5*)	**2**	**4**	**7**
**Benign**	**4** (*4,0*)	**1**	**2**	**1**
FCD	1	0	1	0
CSL	1	1	0	0
Fibroadenoma	1	0	1	0
NAC pCR	1	0	0	1
**Total**	**32** (*23,9*)	**5**	**10**	**17**

*DCIS* Ductal carcinoma in situ, *LCIS* lobular carcinoma in-situ, *FCD* fibrocystic disease, *CSL* complex sclerosing lesion, *NAC pCR* neoadjuvant chemotherapy complete pathologic response

### Micro-CT findings and comparison to single-view specimen mammography

**Fig. 1:**
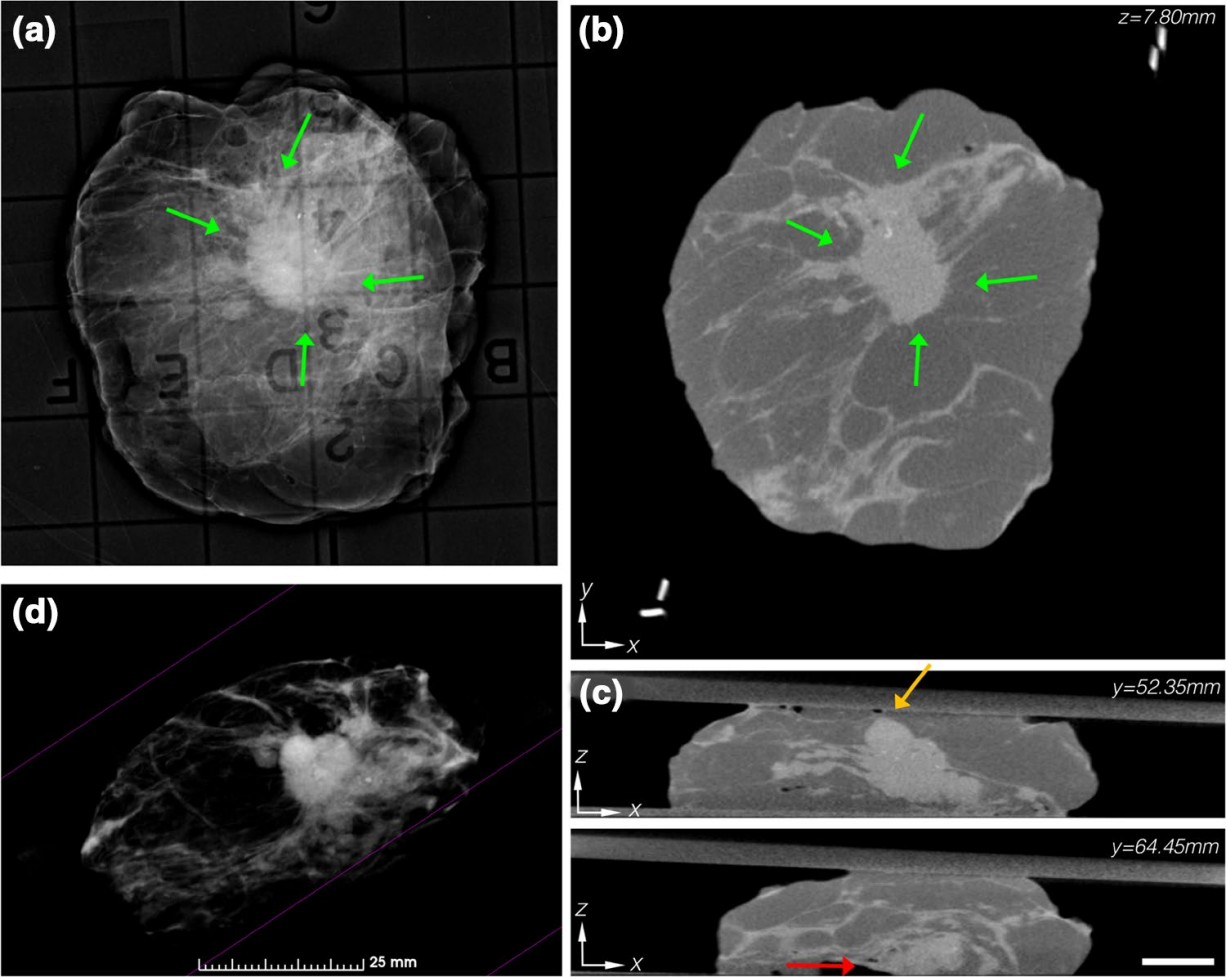
**a** Specimen mammography showing a centrally located IDC + DCIS lesion. **b** Reconstructed micro-CT slice similarly shows a centrally located lesion. **c** Orthogonal micro-CT slices 12.1 mm apart reveal a close (≤ 2 mm) anterior margin (orange arrow) and a pathologically positive deep margin (red arrow), which agreed with the final pathologic diagnosis. Micro-CT scale bar is 1 cm. **d** 3D volume rendering of the specimen, with the adipose tissue thresholded to be transparent, shows the architecture of the lesion and its spiculations (see video in Additional File 2)

In Fig. 1, summary images for a pathologically positive specimen with an IDC + DCIS lesion are shown. In both the single-projection specimen mammogram (Fig. 1a) and the cross-sectional (*x,y*) micro-CT plane (Fig. 1b), a high contrast, highly speculated lesion was centrally located and surrounded by adipose tissue (green arrows). But in an orthogonal (*x,z*) plane (Fig. 1c), the lesion came close to the anterior margin (orange arrow) and touched the posterior margin (red arrow) 12.1 mm apart. This specimen was correctly read as positive, and highlights the ability to resolve lesion involvement spatially with micro-CT relative to a specimen radiograph. Rending of the specimen in Fig. 1d with the adipose tissue thresholded to be transparent reveals the structure of the lesion in 3D (video provided as Supplemental File 2).

**Fig. 2:**
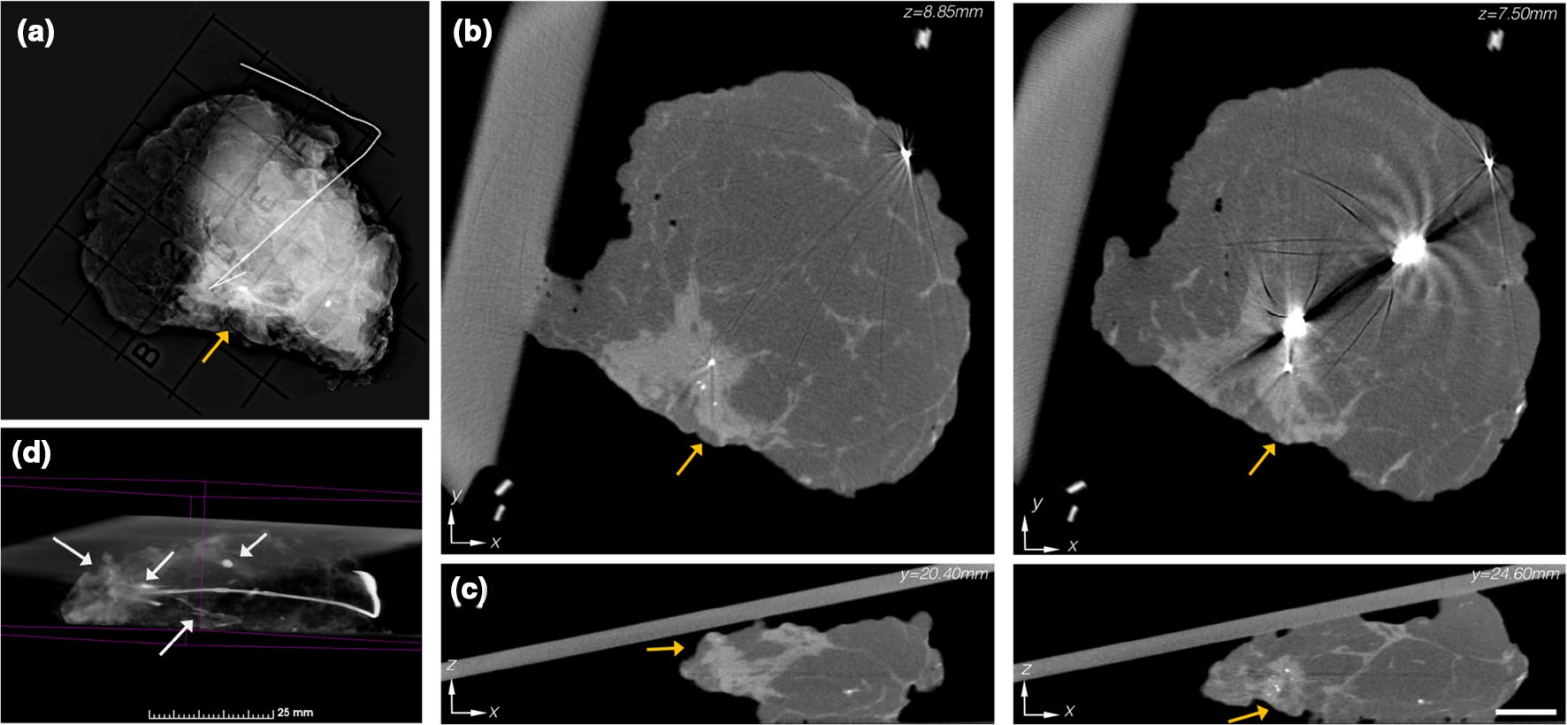
**a** Single-projection specimen mammogram revealing a peripherally located IDC + DCIS lesion (orange arrow). **b** Reconstructed micro-CT slices (1.35 mm apart) similarly depict the peripherally located mass. Notable metal reconstruction artifacts occur because of the localization wire. **c** Orthogonal micro-CT slices (4.2 mm apart) suggest a close peripheral margin, and also indicate a close deep margin, which would not be evident in the single-projection specimen mammogram. Micro-CT scale bar is 1 cm. **d** 3D volume rendering of the specimen, with the adipose tissue thresholded to be transparent, revealed a micro-calcification cluster, a large smooth calcification, tumor location metal clip, and calcified vasculature (white arrows) in the presentation (see video in Additional File 3)

In Fig. 2, summary data for a pathologically negative specimen with an IDC + DCIS lesion are presented. A peripherally located, wire localized lesion with a cluster of micro-calcifications (orange arrows) is evident in the single-projection specimen mammography (Fig. 2a). In cross-sectional (*x*–*y*) micro-CT views of the specimen (Fig. 2b), the lesion can likewise be observed at *z* = 8.85 mm; however, at *z* = 7.50 mm, notable artifacts occur from the wire. In the orthogonal (*x*–*z*) plane, the lesion was read as close to the peripheral margin at *y* = 20.40 mm in agreement with the single-projection specimen mammogram, but at *y* = 24.60 mm the lesion is was also read as close to the deep posterior margin, which was not accessible in the single-projection specimen mammogram. The micro-CT rendering of this specimen (Fig. 4d) resolved the 3D formation and organization of the micro-calcification cluster near the surgical clip and calcified vasculature (white arrows) (video provided as Supplemental File 3).

**Fig. 3:**
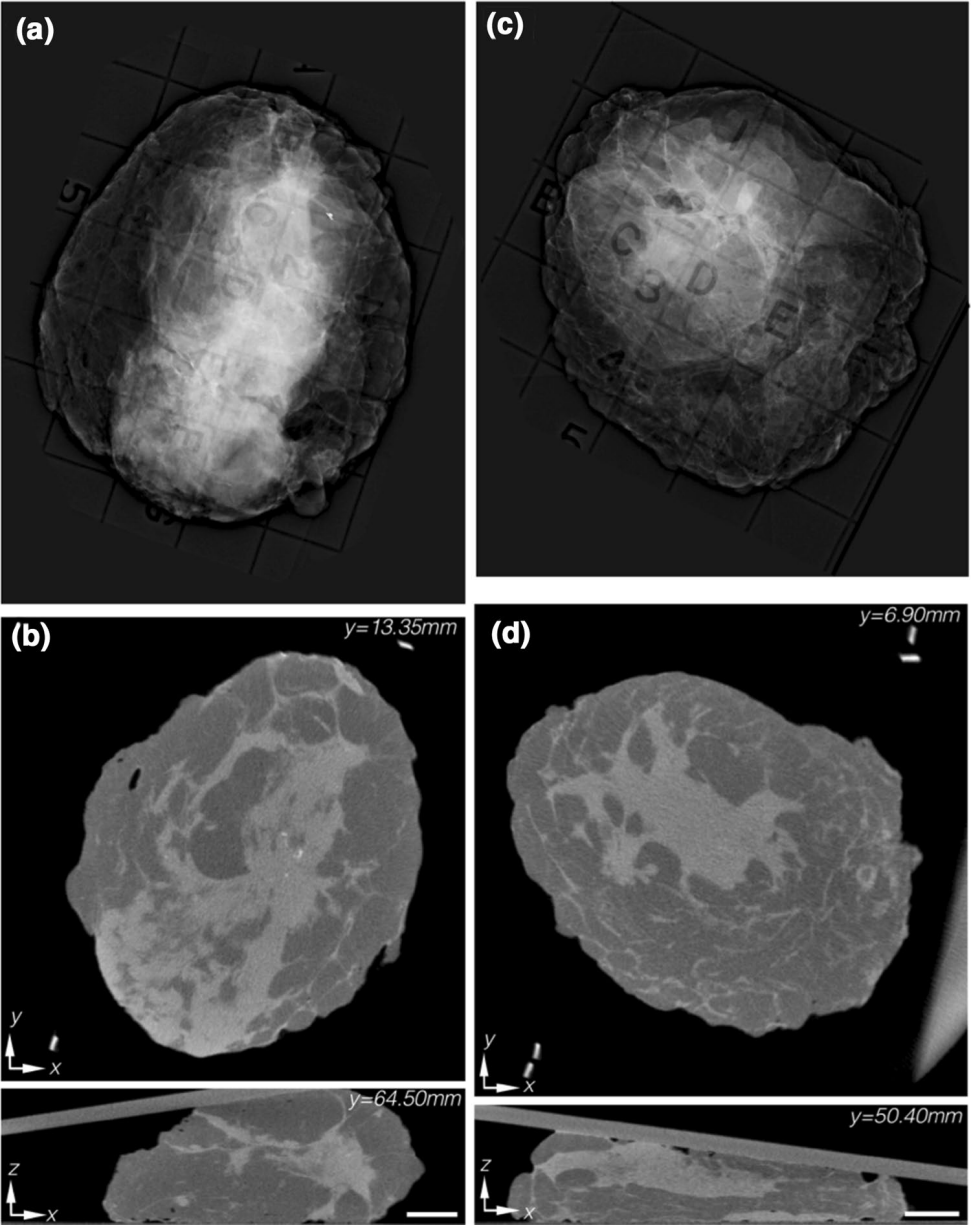
**a** Single-projection specimen mammogram showing a diffuse ILc with poor contrast due to diffuse overlapping structures. **b** Orthogonal reconstructed micro-CT slices resolve the glandular-fat interface in 3D. Pathology confirmed the specimen to have a pathologically positive margin, although it was not detected by micro-CT analysis. **c** Single-projection specimen mammogram showing a centrally located IDC + DCIS lesion. **d** Orthogonal reconstructed micro-CT slices indicate a centrally, but also superficially, located lesion. Pathology confirmed the lesion to be 2.5 mm from the superficial margin, illustrating that micro-CT was unable to resolve the tumor-connective tissue interface. Micro-CT scale bar is 1 cm

In Fig. 3a, b, summary images are presented for a specimen with an ILc lesion. In the single-projection specimen mammogram (Fig. 3a), centrally located bright, diffuse features were present. However, in the orthorgonal micro-CT slices (Fig. 3b), these overlapping structures were resolved and revealed an adipose-glandular tissue interface. Although this specimen did have a pathologically positive margin but was read as negative, the micro-CT was able to resolve the overlapping tissue structures. The single-projection specimen mammogram for another IDC + DCIS lesion is shown in Fig. 3c, and the orthogonal micro-CT slices appear in Fig. 3d. While the lesion seemed to be centrally located in both the single-projection mammogram and the corresponding (*x*–*y*) plane of the micro-CT, the lesion was read as positive on the anterior margin of the *x*–*z* plane. However, the margin was pathologically negative with the lesion 2.5 mm deep, and illustrates a potential limitation of micro-CT resulting from poor tumor to glandular tissue contrast. A summary of orthogonal micro-CT slices through the lesion of each specimen can be found as Supplemental File 4.

### Comparison of micro-CT reading to final pathological diagnosis

Outcomes comparing the micro-CT readings with the final pathological diagnoses are reported in Table 2. Of the four malignant specimens with a pathologically positive margin, two were correctly read, and of the 24 malignant specimens without a pathologically positive margin, 16 were correctly evaluated, yielding a sensitivity, specificity and accuracy of 50% [2/4, CI (7%–93%)], 67% [16/24, CI (45%–84%)], and 64% [18/28, CI (47%–81%)]. While a notable number of pathologically false positives occurred in the malignant micro-CT readings, yielding a positive predictive value of 20% [2/10, CI (4%–44%)], the negative predictive value of 89% [16/18, CI (74%–96%)] was far superior. Of the four benign specimen readings, there was one pathological true positive being a complex sclerosing lesion (CSL) and one pathological false positive being a neoadjuvant chemotherapy lesion with a complete pathologic response. These results represent an initial assessment of the imaging method, as micro-CT scanning of whole BCS specimens is still an investigative modality. The reader was briefed on the micro-CT system, but no official training or guidelines on how to interpret the micro-CT scans was available, given the pre-clinical nature of the technology.

**Table 2:** Comparison of micro-CT reading to pathologic evaluation

	All specimens (*n* = 32)			Only malignant (*n* = 28)		
	Pathologically positive	Pathologically negative	Total	Pathologically positive	Pathologically negative	Total
Positive reading	3	9	12	2	8	10
Negative reading	2	18	20	2	16	18
Total	5	27	32	4	24	28
Sensitivity	60% (15%–95%)			Sensitivity	50% (7%–93%)	
Specificity	67% (46%–84%)			Specificity	67% (45%–84%)	
Accuracy	66% (47%–81%)			Accuracy	64% (47%–81%)	
PPV	25% (12%–45%)			PPV	20% (4%–44%)	
NPV	90% (75%–96%)			NPV	89% (74%–96%)	

*PPV* positive predictive value, *NPV* negative predictive value, *Parenthesis* 95% confidence intervals

### Frequency of margin specific lesion involvement

**Fig. 4:**
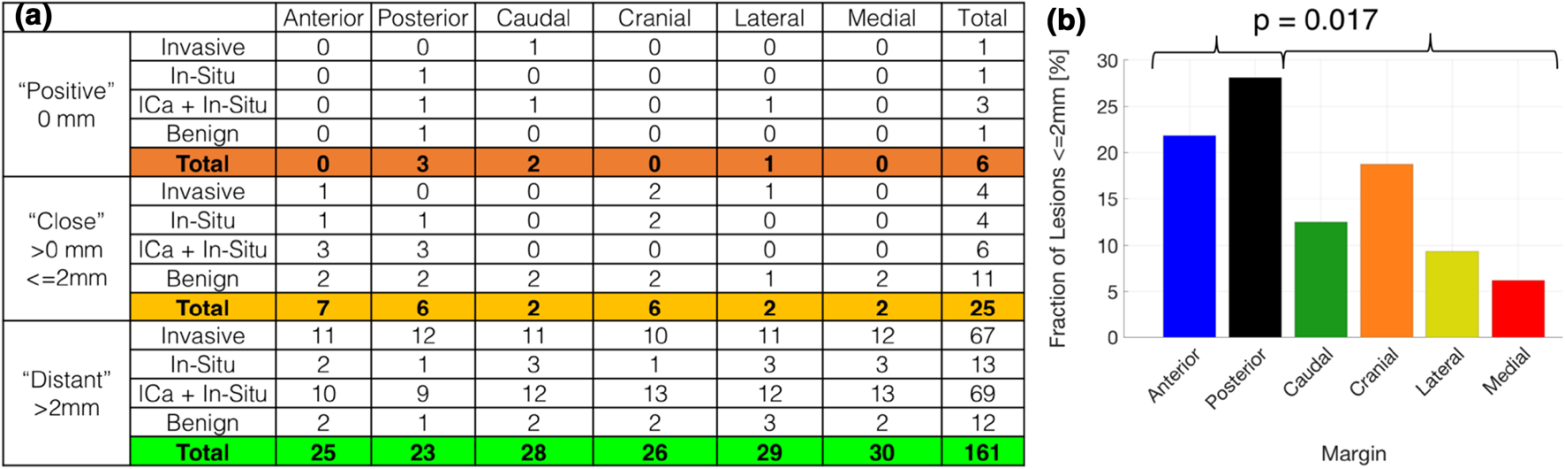
**a** Table summarizing the frequency of pathologically positive, close, and distant lesion involvement for each specific margin. **b** Bar graph of fraction of lesions ≤ 2 mm for each margin, showing a significant increase in close anterior-posterior margins compared to the orthogonal margins. (16/64 ≤ 2 mm, 15/128 > 2 mm, *p* < 0.02). ICA invasive breast cancer

The frequency of pathologically positive (0 mm), close (≤ 2 mm), and distant (> 2 mm) lesions for each of the six margins is tabulated in Fig. 4a. Six margins are listed as positive in total because one positive specimen had two separate positive margins. Similarly, a total of 25 close margins were found from 15 positive and close specimens. While, ICA + In-Situ lesions resulted in the most frequent number of positive and close margins for malignant lesions, this result is not statistically significant, and is limited by a small sample size. The percentage of aggregate positive and close lesions for each margin is plotted in Fig. 4b. Interestingly, anterior and posterior margins had significantly higher frequency of positive and close margins (16/64) compared to their orthogonal margin counterparts (15/128) (*p* = 0.017).

## Discussion

Positive surgical margins after BCS remain a notorious clinical challenge. Wire localization and specimen mammography serve as the primary forms of intra-operative guidance but have led to re-excision rates ranging from 0 to 70% [10]. Microscopic point assessment techniques, such as frozen-section pathology and touch prep cytology, have been used for directed BCS guidance with apparent diagnostic accuracy, but wide-spread adoption has been limited due to increasing labor costs of microscopic sampling over a macroscopic specimen [21]. Accordingly, an advantage of micro-CT demonstrated in this study is its ability to image the entire specimen in 3D, thereby enabling analyses of the complete specimen.

Although modest sensitivity and specificity (50%, 2/4, 67%, 16/24) were reported in this study, significantly more pathologically false positives (8/28) occurred than pathologically false negatives (2/28) (*p* = 0.04) for malignant lesions. While excising unnecessary tissue is less undesirable, it may be less harmful than a second surgical procedure. This notion is highlighted by the proposed practice of undirected whole margin cavity shaving on all BCS procedures, which has recently been investigated [22]. Furthermore, single-projection specimen mammography has reported sensitivity and specificity of 36% and 71% [7], whereas standard-of-care two projection specimen mammography has yielded sensitivities and specificities of 54.6% and 87.8% [23], and more recently 58.5% and 91.8% [24]. While the preliminary micro-CT results presented here suggest improved sensitivity and comparable specificity to single-projection mammography, and comparable sensitivity but inferior specificity to two projection mammography, a prospective, fully powered, head to head study of micro-CT against standard-of-care two projection specimen mammography is needed before the performances of the two modalities can be accurately compared. Additionally, the reading of micro-CT specimen scans was not optimized and no training was provided, the latter would benefit from an atlas of micro-CT scans of BCS specimens but none currently exists. The data presented here could serve as the starting point for a training set for the presentation of various pathologies in micro-CT. Nevertheless, reading individual cross-sectional planes of specimens in the projection views micro-CT appears to offer valuable information on lesion location.

This inceptive study provides initial evidence suggesting that micro-CT specimen scanning offers potential diagnostic value, which could be quantified in a larger prospective study. While this study demonstrated the ability to detect in-situ disease from the analysis of clustered micro-calcifications, a larger sample size is needed to more reliably quantify the accuracy of detecting invasive versus in-situ disease. A notable limitation of micro-CT was its lack of contrast between tumor-glandular tissue, which resulted in poor diagnosis of tumor-glandular interfaces. While a lower kVp could increase image contrast, this would require a longer exposure time to maintain photon counts and SNR. Alternatively, dual energy contrast enhancement could be envisioned to increase contrast, but this would require two scans at different energies as well as the injection of a contrast agent [25]. Another limitation was the lack of radiologic training available prior to the study. In future, prospective studies, the data presented in this manuscript could be used as a radiologic atlas and training data set, which could potentially improve reader performance. Another limiting factor was the presence of metal streaking artifacts arising in reconstructions of specimens containing localization wires. In Tang et al., this difficulty was mitigated by first performing a single-projection image to confirm the wire and surgical clip were excised, then removing the wire from the specimen, and performing the full micro-CT scan [14]. However, this approach eliminates the wire and clip which are used regularly as lesion landmarks during subsequent gross analysis in Pathology. An alternative solution would be to implement a metal artifact reduction algorithm, such as segmenting the metal, manually setting its known attenuation coefficient, and performing an iterative reconstruction [26, 27]. Finally, it should be noted that the gold-standard histopathological margin analysis, which samples only a subset of the margin chosen through gross dissection and evaluation, has shown to only weakly correlate with the actual presence of disease in the lumpectomy cavity, with reported accuracies as low as 64.9% and up to 20% of pathologically negative margins presenting residual disease [28, 29]. This raises the possibility that micro-CT may be able to detect disease invisible during gross dissection and subsequent pathological analysis, and motives the need for more spatially exact correlations between micro-CT readings and pathological confirmation in future studies.

In summary, the results reported here demonstrate the feasibility of using micro-CT to provide full volume 3D microscopic reconstructions of lumpectomy specimens that highlight spatially-resolved features not evident in conventional 2D projection radiography. Furthermore, the study offers an initial measure of the correlation between micro-CT analysis of whole BCS specimens and current gold-standard histopathology. Micro-CT should continue to be investigated as a potential BCS guidance tool because of its rapid acquisition and volume rendering capabilities.

## Electronic supplementary material

Below is the link to the electronic supplementary material.

**Margin-Lesion Distances**. A complete summary of the distance to lesional involvement for each margin of each specimen. (PNG 1129 KB)

**Volume Rendering Video 1**. A 3D volume rendering of an invasive ductal carcinoma specimen with DCIS. The adipose tissue has been thresholded to be transparent, revealing the 3D architecture of the centrally located lesion and its spiculations. (MP4 9553 KB)

**Volume Rendering Video 2**. A 3D volume rendering of an invasive ductal carcinoma specimen with DCIS. The adipose tissue thresholded to be transparent, revealing a peripherally located micro-calcification cluster and lesion, near the tumor localization wire and clip. Additionally, a large smooth calcification and calcified vasculature can be seen away from the lesion. (MP4 5654 KB)

**CT-Slice Montage of All Casse**. A montage of illustrative orthogonal micro-CT slices through each lesion of all 32 specimens. (PNG 3591 KB)

## Abbreviations

BCS: Breast conserving surgery
Micro-CT: Micro-computed tomography
2D, 3D: Two, three dimensional
ICa: Invasive breast cancer
IDc: Invasive ductal carcinoma
ILc: Invasive lobular carcinoma
DCIS: Ductal carcinoma in-situ
LCIS: Lobular carcinoma in-situ
FCD: Fibrocystic disease
CSL: Complex sclerosing lesion
