# Supplementary figures and images for: Micro-computed tomography enables rapid surgical margin assessment during breast conserving surgery (BCS): correlation of whole BCS micro-CT readings to final histopathology
Source: Breast Cancer Res Treat. 2018 Sep 17;172(3):587–95. doi: 10.1007/s10549-018-4951-3 (PMC6245085; doi:10.1007/s10549-018-4951-3)

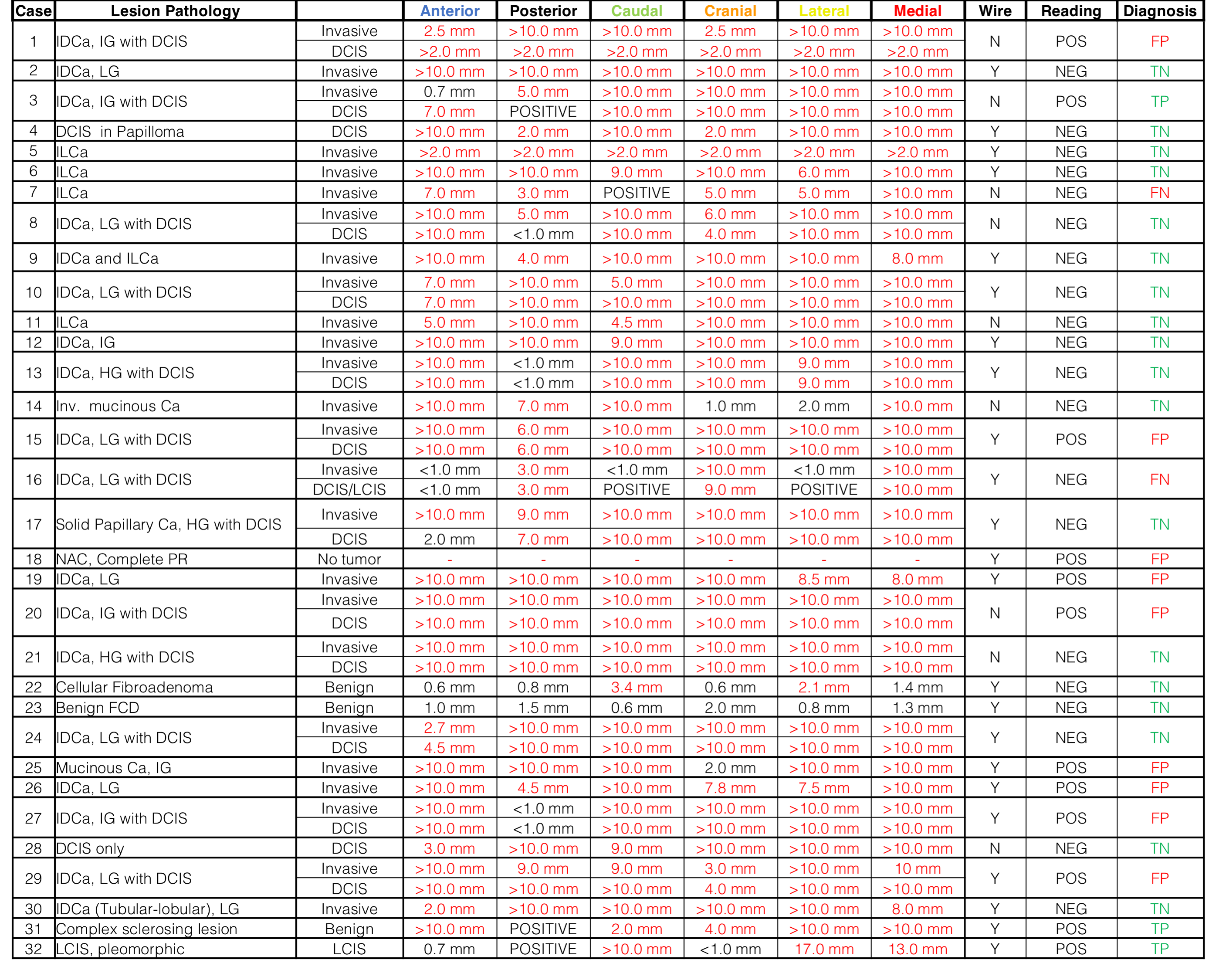

Supplement: Supplementary file 1 — Margin-Lesion Distances. A complete summary of the distance to lesional involvement for each margin of each specimen. (PNG 1129 KB) [file 10549_2018_4951_MOESM1_ESM.png]

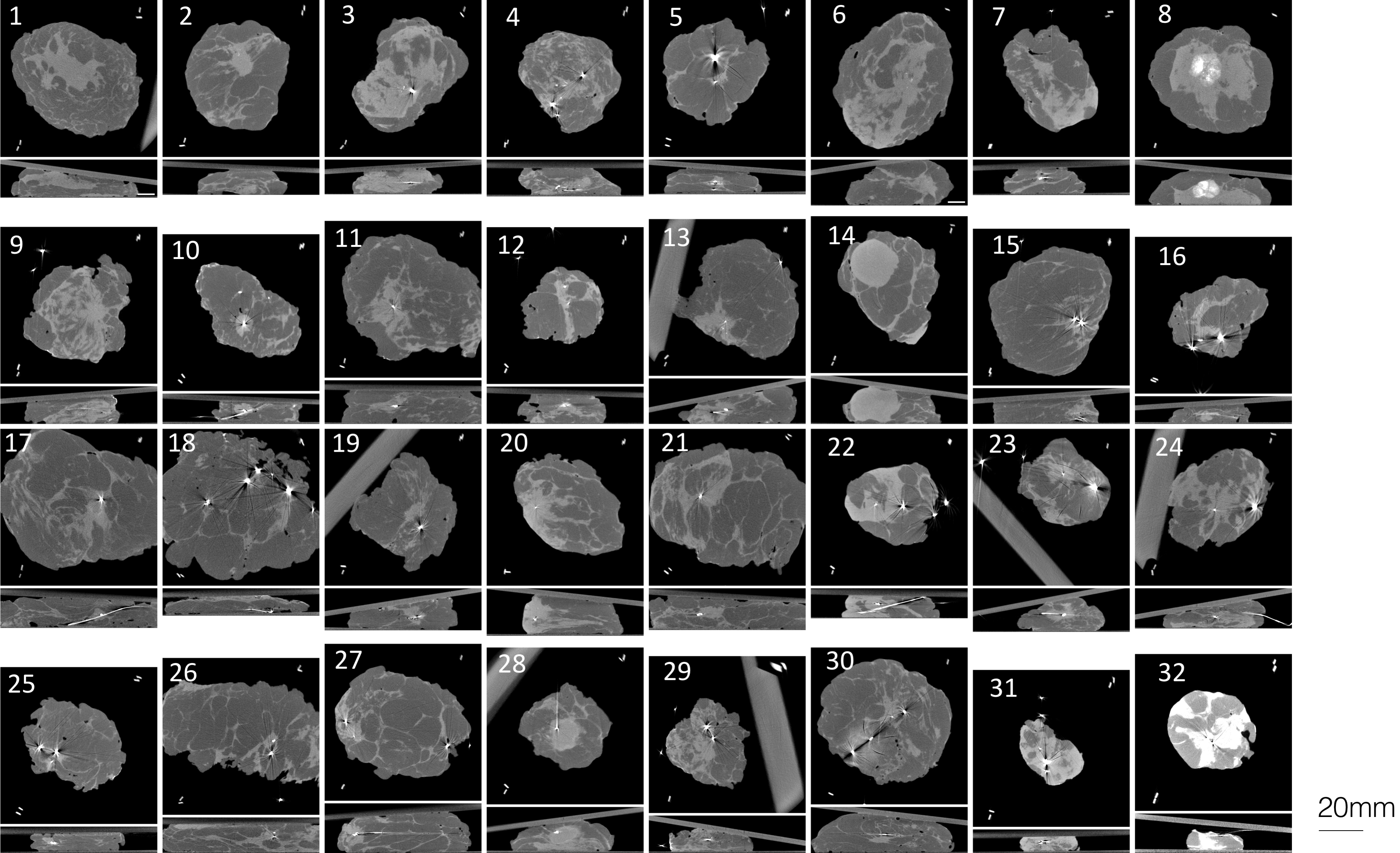

Supplement: Supplementary file 4 — CT-Slice Montage of All Casse. A montage of illustrative orthogonal micro-CT slices through each lesion of all 32 specimens. (PNG 3591 KB) [file 10549_2018_4951_MOESM4_ESM.png]
